# Computerised Cardiotocography Analysis for the Automated Detection of Fetal Compromise during Labour: A Review

**DOI:** 10.3390/bioengineering10091007

**Published:** 2023-08-25

**Authors:** Lochana Mendis, Marimuthu Palaniswami, Fiona Brownfoot, Emerson Keenan

**Affiliations:** 1 Department of Electrical and Electronic Engineering, The University of Melbourne, Parkville, VIC 3010, Australia; palani@unimelb.edu.au (M.P.); e.keenan@ieee.org (E.K.); 2 Obstetric Diagnostics and Therapeutics Group, Department of Obstetrics and Gynaecology, The University of Melbourne, Heidelberg, VIC 3084, Australia; fiona.brownfoot@unimelb.edu.au

**Keywords:** fetal compromise, intrapartum fetal monitoring, cardiotocography, fetal heart rate, artificial intelligence

## Abstract

The measurement and analysis of fetal heart rate (FHR) and uterine contraction (UC) patterns, known as cardiotocography (CTG), is a key technology for detecting fetal compromise during labour. This technology is commonly used by clinicians to make decisions on the mode of delivery to minimise adverse outcomes. A range of computerised CTG analysis techniques have been proposed to overcome the limitations of manual clinician interpretation. While these automated techniques can potentially improve patient outcomes, their adoption into clinical practice remains limited. This review provides an overview of current FHR and UC monitoring technologies, public and private CTG datasets, pre-processing steps, and classification algorithms used in automated approaches for fetal compromise detection. It aims to highlight challenges inhibiting the translation of automated CTG analysis methods from research to clinical application and provide recommendations to overcome them.

## 1. Introduction

Despite ongoing advances in maternal and fetal healthcare, over 2 million babies are stillborn worldwide every year [1,2,3]. Depending on global region, between 6% and 49% of these stillbirths occur during labour compared to the antepartum period [1]. Many of these stillbirths could be prevented with monitoring during pregnancy [4] and timely obstetric intervention for complications during childbirth [5]. The most common cause of stillbirth globally is intrapartum asphyxia [6], which typically occurs when mechanical pressure during labour limits blood flow to the baby, resulting in a reduction in oxygen delivery to the fetus. When oxygen levels are critically low, the fetus changes its metabolism from aerobic to anaerobic, resulting in hydrogen ion formation in tissues, known as acidosis [7]. In addition to total oxygen deprivation, which may result in stillbirth, this build-up of acid is extremely damaging, especially to neuronal cells in the fetal brain [8]. This can result in short- and long-term brain injury, leading to complications, including cerebral palsy and intellectual impairment for surviving infants [9]. For this reason, the early detection of fetal compromise is extremely important so that clinicians can intervene before irreversible harm occurs.

Due to these hypoxic events triggering the fetal sympathetic and parasympathetic nervous systems, variations in the fetal heart rate (FHR) can be monitored to detect signs of fetal compromise [8,10,11]. The primary source of FHR surveillance in the early half of the twentieth century was via the use of intermittent auscultation, and in the late 1960s, with the development of ultrasound technology, continuous cardiotocography (CTG) was introduced for fetal monitoring [12]. CTG extracts both the FHR and uterine contractions (UC) simultaneously, allowing for the continuous assessment of both signals.

FHR and UC are typically extracted using either invasive or non-invasive methods. The most widely used method is the non-invasive Doppler ultrasound and tocodynamometer, which uses two external transducers placed on the mother’s abdomen. These signals, however, are subject to signal dropout due to fetal and maternal movements, and their signal quality deteriorates with an increase in maternal body mass index (BMI) [13]. Therefore, more accurate readings are sometimes obtained using invasive monitoring methods such as the direct fetal electrocardiogram (fECG) acquired via a fetal scalp electrode for FHR and intrauterine pressure catheter for UC [14,15]. Despite their benefits, these invasive modes of measurement have an increased risk of infection and can only be used by rupturing the membranes, making them suitable for use during labour only [14,16]. As a result, new methods such as the non-invasive fetal electrocardiogram and electrohysterogram are being introduced to overcome the shortcomings of their predecessors [17]. Throughout this paper, we use the term CTG to refer to any method that measures both FHR and UC simultaneously.

Since its introduction, CTG has become a routinely used technique in clinical practice for fetal monitoring in labour. However, despite the wide use of CTG, it has not been able to show a significant reduction in fetal mortality [18,19,20]. In practice, CTG recordings are visually evaluated and assessed based on guidelines, leading to substantial inter- and intra-observer disagreement among clinicians [21,22,23]. Furthermore, the high false positive rate of visual CTG evaluation is cited as one of the reasons behind the increasing rate of caesarean sections (CS) and unnecessary operative deliveries [24]. Therefore, there is an unmet clinical need for a more reliable, reproducible and objective CTG evaluation system.

As CTG evaluation provides an indirect measure of fetal asphyxia, a more direct and quantitative measure of fetal asphyxia is pH. To evaluate pH, a blood sample is taken from either the fetal scalp during labour or the umbilical cord immediately after birth [25]. The pH value measures the level of fetal acidemia arising from the dispersion of accumulated lactic acid in the fetal circulation [26]. The base deficit (BDecf) is another parameter determined from the umbilical cord blood, which is closely linked to fetal asphyxia. pH values below 7.05 and BDecf values above 10 mmol/L are strongly related to adverse neonatal outcomes [26,27]. Another metric used to assess fetal compromise is the Apgar score, which evaluates the physical condition of the baby shortly after delivery, where the 5-min Apgar score has been shown to be a predictor of the risk of neonatal death [28]. Therefore, umbilical artery pH, BDecf and Apgar are common clinical endpoints used to define fetal compromise.

With the availability of digital CTG records and clinical endpoints defining the presence of fetal asphyxia, alternative computerised data-driven approaches have been proposed to reduce misinterpretation and improve the accuracy of CTG evaluation. Initially, these methods tried to approximate expert interpretations based on guidelines provided by the International Federation of Gynaecology and Obstetrics (FIGO) by analysing baseline variability, accelerations and decelerations of the FHR [29]. The Dawes–Redman System and the Porto System are two systems that first adopted these features for automated CTG analysis in clinical practice [30,31], followed by the INFANT system [32,33]. Thereafter, additional FHR features from time, frequency and non-linear domains were introduced in addition to the morphological features. A CTG analysis prototype system called the OxSys [34] based on one of the non-linear FHR features, decelerative capacity, was recently introduced to analyse and trigger real-time alerts to clinicians.

Concurrent to these clinical methods, FHR features were also utilised in data-driven approaches to train machine learning (ML) algorithms to detect fetal compromise [35,36]. Subsequently, modern data-driven methods like deep learning (DL) algorithms were proposed, which independently learn information from the raw CTG waveforms minimizing the need for human-guided feature extraction and selection [37]. However, comparisons between the performance of these novel data-driven methods are difficult as different outcome measures and different case selection criteria have been used across studies. The most commonly used biomarker in differentiating CTGs with fetal compromise in these computer-based methods is umbilical artery pH with a threshold set at 7.05 [35,37,38,39,40]. Other alternative cut-off pH values such as 7.10 [41,42], 7.15 [43,44,45,46] and 7.20 [47] have also been employed by previous studies. Nevertheless, these algorithms have shown the potential to improve the sensitivity of detecting fetal compromise but have not yet been translated into widespread clinical use.

This narrative review aims to synthesise existing literature on computerised cardiotocography analysis for fetal compromise detection, with a particular focus on automated data-driven techniques and a comprehensive overview of their associated challenges. This complements recent reviews associated with the physiology of FHR variability during labour [48], signal processing techniques for FHR analysis [49,50,51], challenges for developing AI techniques for FHR monitoring [52,53], and computerised CTG analysis in clinical practice [54]. One of the primary focuses of this paper is to identify a standardised approach for CTG segment length, pre-processing, and definition of outcomes for fetal compromise to enable the effective comparison of data-driven analysis methods.

**Figure 1:**
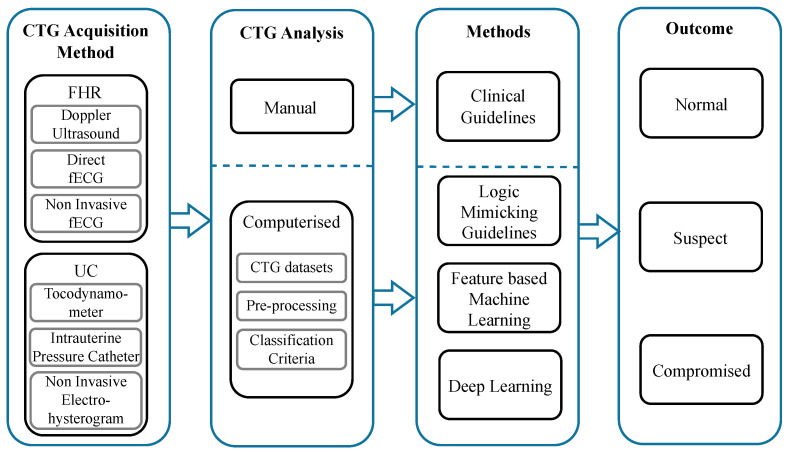
Summary of the computerised CTG analysis process for fetal compromise detection. The process begins with the CTG acquisition methods for fetal heart rate and uterine contractions. The output of these signals is then analysed by two methods: visual inspection or a computerised approach. The prior is based on clinical guidelines, and the latter uses artificial intelligence to analyse the FHR. The outcome of the analysis is to predict the status of the baby as normal, suspect or compromised.

To achieve this, we first provide a brief overview of the physiological basis for CTG monitoring and then summarise FHR and UC monitoring technologies and associated challenges with data extraction. Then, we provide an overview of the current state of the existing computerised CTG systems in clinical practice. Next, we provide a summary of public and private CTG datasets, followed by a review of pre-processing techniques reported in the literature. Following this, we comprehensively review the data-driven computerised approaches for classifying fetal compromise, from proposed methods based on feature-based machine learning to the latest advancements in deep learning. Finally, the current obstacles that limit progress within this field are identified, and recommendations for future research are presented. A summary of the process for the computerised CTG analysis of fetal compromise detection and the core topics addressed in this review are shown in Figure 1.

## 2. Physiological Basis for CTG Monitoring during Labour

The fetal heart rate is regulated by the autonomic nervous system, primarily through sympathetic and parasympathetic influences [8,10,55]. During labour, the FHR is influenced by various factors such as changes in oxygenation as a result of insufficient placental perfusion, fetal movement and umbilical cord compression. These factors give rise to distinct FHR patterns, which are typically categorised as changes in baseline, variability, accelerations and decelerations. The baseline is the average FHR, excluding accelerations and decelerations. The variability is the fluctuation of the FHR around the baseline, reflecting the balance between the sympathetic and parasympathetic activity. Accelerations are transient increases in FHR above the baseline, indicating a healthy fetal response to stimuli. Decelerations are transient decreases in FHR below the baseline, which can be either physiological or pathological depending on their shape, timing, duration, and association with uterine contractions [11,56].

Uterine contractions also play a central role during labour, resulting from the coordinated activity of uterine smooth muscles. Oxytocin stimulates contractions’ frequency and intensity, while prostaglandins contribute to cervical ripening [57]. These contractions can impact FHR through changes in uteroplacental perfusion or umbilical cord compression. Intense or frequent UCs may compromise fetal oxygenation by limiting blood flow, potentially leading to FHR decelerations [55]. Alternatively, UC-induced stress can stimulate fetal sympathetic activity, resulting in FHR accelerations [26,57]. Therefore, the simultaneous monitoring of UC and FHR patterns using CTG can aid clinicians in assessing both labour progression and fetal status.

## 3. Fetal Heart Rate and Uterine Contraction Monitoring Technologies

**Figure 2:**
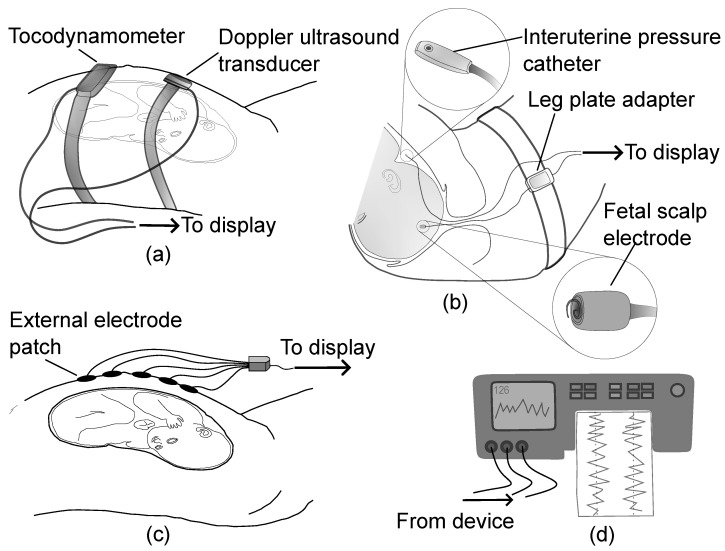
An overview of fetal monitoring technologies for capturing FHR and UC showing (**a**) Doppler ultrasound and tocodynamometer, (**b**) direct fetal electrocardiogram using scalp electrode and intrauterine pressure catheter, (**c**) non-invasive fetal electrocardiogram and electrohysterogram using external electrodes, and (**d**) the display unit for monitoring the extracted data in digital or paper format.

Although the FHR and UC signals produced across CTG monitoring systems are displayed in a consistent format, they may be acquired using different modalities that have different accuracy profiles, benefits and limitations. These signals are critical for computerised CTG analysis, and problems with input signal quality can lead to incorrect predictions in subsequent steps. To highlight the importance of this area, this section provides a review of various technologies utilised to extract FHR and UC signals. An overview of fetal monitoring technologies for capturing FHR and UC is shown in Figure 2.

### 3.1. Fetal Heart Rate Monitoring Technologies

#### 3.1.1. Doppler Ultrasound

Doppler ultrasound is a non-invasive form of monitoring the fetal heart rate. It comprises an ultrasound transducer strapped to the maternal abdomen, which emits an ultrasound wave and measures the reflected waveforms resonated with the fetal cardiac structures. Fetal heartbeats are then approximated using signal modulation and autocorrelation techniques [58]. Due to the nature of ultrasound monitoring, the extracted FHR recordings are prone to signal losses caused by the movements of the baby, maternal movements and transducer displacement. Signal artefacts also occur particularly due to accidental monitoring of the maternal heart rate [11] and half or double counting fetal heart rate values when using autocorrelation techniques for FHR approximation [59]. These noises are typically seen more often during the second stage of labour [14]. The quality of the FHR measured using the Doppler ultrasound method is also hampered by maternal factors like increased BMI [60]. In terms of reliability, the mean positive percent agreement of the Doppler ultrasound compared to fetal scalp electrode during labour is between 62 and 73% [61,62]. Despite these limitations, Doppler ultrasound has been widely used in monitoring FHR during labour since its introduction in the 1960s. According to a United States (U.S.) national survey, 98% of mothers who gave birth in a U.S. hospital from 2011–2012 underwent Doppler ultrasound CTG monitoring at some point during labour [63].

#### 3.1.2. Direct Fetal Electrocardiogram

The direct fetal electrocardiogram is an invasive method where a fetal scalp electrode is inserted through the mother’s cervix and attached to the presenting part of the fetal scalp. It enables the measurement of the time difference between two consecutive heartbeats by identifying the R waves of the direct fECG [11,64], allowing for a more accurate beat-to-beat FHR to be obtained. However, the electrode can only be placed when the amniotic sac around the baby has ruptured and the cervix is dilated [64]. This limits its usage to monitoring FHR only during labour. Further, it is typically avoided in fetuses under the gestational age of 32 weeks and in mothers who are infected by blood-borne viruses [11]. Additionally, by using this method in clinical practice there is an increased risk of infections, injury, and bruising of the fetal scalp. This method is also more expensive to use as the electrodes are disposable [11]. However, the direct fECG monitoring of FHR through a scalp electrode is the most accurate method for fetal surveillance during labour [14,65].

#### 3.1.3. Non-Invasive Fetal Electrocardiogram

A desire to overcome the limitations of Doppler ultrasound and the invasiveness of the direct fECG has led to the introduction of an alternate method of monitoring called the non-invasive fetal electrocardiogram (NI-fECG). This method uses several electrodes placed on the maternal abdomen to record the electrical activity of the fetal heart which is used to extract the FHR rhythm. The main challenge faced by this method is the low signal-to-noise ratio of the fetal ECG compared to that of maternal ECG and the presence of different dielectric media between the sensors and the electrodes [66,67,68,69]. However, the NI-fECG method provides more reliable FHR readings compared to Doppler ultrasound in labour with a mean positive percent agreement between 81–83% than the fetal scalp electrode [61,62], with no significant decrease in performance for high BMI patients [13]. However, there are limitations to these studies, where agreement analysis was only performed in a subset of recording segments where fetal heart rate extraction was successful. The percentage of successful recording segments varied between Doppler and NI-fECG methods, as well as there were variations in the number of participants included for analysis during the second stage of labour. As such, further studies are required to demonstrate that NI-FECG offers comparable performance to direct fetal ECG scalp monitoring.

### 3.2. Uterine Contraction Technologies

#### 3.2.1. External Tocodynamometer

An external tocodynamometer (TOCO) is a non-invasive strain gauge placed over the maternal abdomen to monitor uterine activity. This device provides information on the frequency and approximate duration of uterine contractions but not their absolute intensity [16,70]. This method can be used both before and during labour but suffers from periods of signal loss arising from misalignment following maternal movements. Furthermore, performance reduces with increasing BMI [71]. Nevertheless, TOCO is the most commonly used modality to monitor uterine contractions as it is non-invasive and simple to use [70].

#### 3.2.2. Intrauterine Pressure Catheter

An intrauterine pressure catheter (IUPC) is an invasive device placed inside the amniotic space during labour to monitor uterine contractions. This method is unaffected by maternal position and obesity and provides the most accurate measurement of the frequency of contractions as well as their strength and duration [15]. Therefore, it is considered the current gold standard for measuring UC. However, it requires the rupture of the membranes and carries the risk of infection and other complications, which limit its widespread use [72].

#### 3.2.3. Non-Invasive Electrohysterogram

The electrohysterogram is a promising non-invasive technique that uses abdominal electrodes to measure uterine electrical activity and derive uterine contractions [73]. The main benefits of this method are that it gives a reliable measurement of the contractions even with obese women compared to TOCO [16,71] and unlike IUPC can be used for continuous UC monitoring in both pregnancy and during labour.

## 4. Computerised CTG Analysis Systems in Clinical Practice

The first computerised CTG analysis system was introduced by Dawes and Redman [30] by analysing the morphology of the FHR to alert clinicians of the risk of pathological outcomes during the antenatal period. In the same year, the Porto system was introduced, which quantitatively adapts the FIGO guidelines for automated CTG analysis during labour [31]. Later, this system was commercialised as SisPorto [74] and Omniview-SisPorto (Speculum, Lisbon, Portugal) [75]. The latest version of this system, SisPorto 4.0 [76], incorporates the FIGO’s 2015 guidelines and provides real-time alerts to features that require attention or clinical intervention. INFANT (K2 Medical Systems, Plymouth, UK) is another decision support system that analyses the FHR and UC patterns to provide alerts to help clinicians during labour. Recently, a new prototype system primarily based on the decelerative capacity of FHR called the OxSys was introduced to analyse and trigger real-time alerts to clinicians [34].

Most of these systems have undergone years of work, rigorous testing, and clinical validation through randomised control trials (RCTs). The largest RCT on 46,042 women evaluated the INFANT system and concluded that the incidence of poor neonatal outcomes was the same regardless of using the decision support system, 0.7% in both groups (adjusted risk ratio 1.01, 95% CI:0.82–1.25) [33]. Another RCT on 7730 patients evaluated continuous central fetal monitoring by computer analysis and real-time alerts of the Omniview-SisPorto 3.5 system with visual analysis and concluded that while both study arms reported lower than expected rate of newborn metabolic acidosis, no significant reduction in the metabolic rate or obstetric intervention was achieved using computerised analysis (relative risk 0.69, 95% CI:0.36–1.31) [77]. Two recent meta-analyses also concluded similar findings that computerised CTG analysis did not improve neonatal outcomes compared to conventional evaluation [78,79]. In another study, a retrospective database of 22,790 women in labour was evaluated using the prototype OxSys 1.5 system. They found that the OxSys system potentially increased the sensitivity of fetal compromise detection (43.3% vs. 38.0% for severe, *p* = 0.3 and 36.1% vs. 31.0% for moderate, *p* = 0.06) while reducing the false positive rate (14.4% vs. 16.3%, *p* < 0.001) compared to conventional clinical diagnosis [34]; however, the sensitivity increase was not statistically significant.

## 5. CTG Datasets

In order to develop new data-driven methods for CTG analysis during labour, it can be seen that large datasets are necessary to capture uncommon clinical outcomes. CTG recordings are typically stored by hospitals in paper format or as digital records in electronic health systems. Research groups with access to digitally accessible records have proposed a range of computer-based CTG analyses with varying dataset sizes, data sampling frequencies, and outcome measures. Nevertheless, there are only two CTG datasets currently available for public access: (1) The University of California, Irvine (UCI) Machine Learning repository CTG dataset, consisting of 2126 records, each with 23 features of FHR and UC signals classified into three classes of normal, suspect and pathological by three expert obstetricians [80]; and (2) The Czech Technical University and University Hospital in Brno (CTU-UHB) dataset, consisting of 552 CTG records with raw FHR and UC signals [81]. It is important to note that the UCI dataset does not contain the raw FHR and UC signals. Additionally, the Lyon dataset [38] and the Oxford dataset are used in the literature [37], but these are not publically available. An overview of these four frequently used datasets is shown in Table 1.

## 6. FHR Pre-Processing Techniques

**Figure 3:**
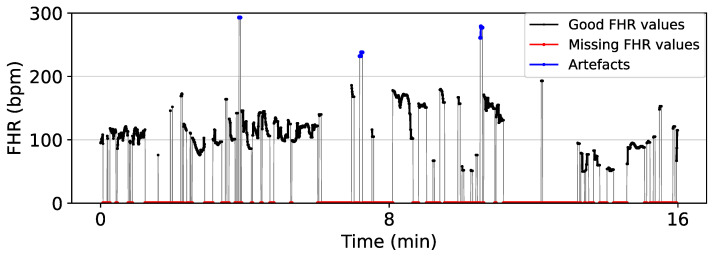
Segment of CTG 1087 from CTU-UHB showing the artefacts and missing values of a raw FHR signal. FHR values in the range 50–200 bpm are considered good values, while FHR values below 50 bpm and above 200 bpm are considered outliers (artefacts). Zero values represent the missing FHR values.

In routine practice, the FHR is monitored through Doppler ultrasound CTG or direct fetal scalp electrode, or sometimes a combination of both. For example, FHR records of the CTU-UHB dataset were acquired by a mixture of Doppler ultrasound CTG and direct fECG [81]. These signals are contaminated by noises composed of artefacts and periods of missing FHR values (denoted by zeros) as shown in Figure 3. Fetal/maternal movements and the displacement of the transducers add noise to Doppler ultrasound CTG monitoring, while vaginal examinations and maternal pushing contribute to noise in CTG monitoring by direct fECG [14]. Generally, for Doppler ultrasound-based CTG, median signal loss is reported to be between 5 and 8% for the first stage of labour and between 9 and 20% for the second stage of labour. The direct fetal scalp electrode demonstrates lower median signal loss between 0.8 and 1% and 3 and 4% in the first and second stages of labour, respectively [14,82,83]. For the above reasons, raw FHR data require pre-processing before analyzing with computer-based methods like machine learning because low-quality data prevent stability and convergence in the learning process [84]. Hence, pre-processing is a crucial step in the automatic evaluation of FHR signals and generally involves the following five steps, which may be performed in differing order:

1. Segment Selection
  In clinical practice, labour is divided into three stages: Stage I is where the cervix starts dilating (<10 cm) and frequent contractions occur; Stage II is the period from when the cervix is fully dilated (10 cm) to when the baby is born; Stage III starts after the baby is born and continues until delivery of placenta and membranes. In the segment selection step, a signal segment with sufficient quality that is closer to the delivery of the baby is typically selected as this is representative of the level of asphyxia and correlates best with the cord pH at birth. Unfortunately, the FHR signal is typically most affected by noise and artefacts at this point of labour [85]. FIGO guidelines require the signal loss to be less than 20% for a signal to be acceptable for evaluation [14]. Different prior studies have selected signal lengths varying from 10–60 min before birth to analyse, whilst others simply select the segment of trace during a certain stage of the labour [44].
2. Artefact Removal
  A typical baseline heart rate of a normal fetus varies between 110 bpm and 160 bpm, and accelerations or decelerations occur when amplitude varies 15 bpm above or below the baseline lasting for more than 15 s respectively [11]. In the artefact removal step, values below 50 bpm and above 200 bpm are typically considered outliers and removed [36]. In some studies, consecutive missing values of more than 15 s (long gaps) are removed from the analysis [36,40,86]. For others, when the difference between two adjacent FHR values exceeds 25 bpm, the corresponding signal segment from the previous FHR value to the next stable segment is considered unstable and removed [45,85]. A stable segment is a signal segment with five consecutive FHR values having a difference of less than 10 bpm between them [31].
3. Signal Interpolation
  The signal interpolation step employs techniques like linear [87] and spline [35] interpolation to fill the missing FHR values created from the previous steps. Generally, the interpolation is performed for gaps < 15 s, and the gaps > 15 s in the FHR are either skipped or removed in subsequent feature extraction and deep learning training processes [35,39,86]. In linear interpolation, these missing gaps are approximated using the slope of the data points on either side of the gap. Spline interpolation uses a set of low-degree polynomials called a spline to estimate the missing gaps to make the signal smoother and continuous. When polynomials of degree 3 are used in the spline, the resulting interpolation is called cubic spline interpolation. The Hermite spline interpolation uses polynomials defined by the values and the derivatives at the endpoints of the corresponding interval to estimate the missing values.
4. Downsampling
  A typical fetal heart beats less than 3 times per second (<180 bpm), making some data of the original FHR signals sampled at 4 Hz redundant [46,87]. Therefore, in this step, the FHR signal is sometimes downsampled to reduce the computational complexity and memory needed to process the input signals. For example, only 900 values are required to represent a 60 min FHR signal at 0.25 Hz, compared to the 14,400 values required for the same signal at 4 Hz.
5. Smoothing and Detrending
  In some works, the final step in pre-processing was to smooth the FHR signal using a median filter [44,88] or detrend the signal before using non-linear signal processing techniques [44,89].

**Figure 4:**
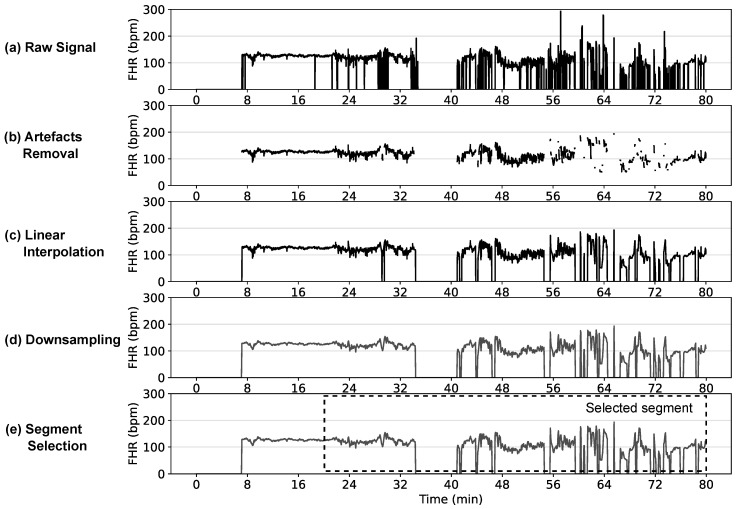
Typical pre-processing steps on a FHR signal showing: (**a**) raw FHR signal at original 4 Hz sampling rate; (**b**) artefact removal of FHR values outside 50–200 bpm and non-physiological values with variation among adjacent FHR values > 25 bpm; (**c**) linear interpolation to fill the missing gaps—only gaps < 15 s are interpolated, the gaps > 15 s are shown as zeros, which will be either skipped or removed in subsequent feature extraction or classification stages; (**d**) downsampled signal at 0.25 Hz; and (**e**) last 60 min segment selected for analysis.

In addition to these conventional and straightforward steps, more sophisticated methods also have been proposed for FHR noise removal and the recovery of missing samples [90,91]. An example of typical pre-processing steps applied to an FHR signal is shown in Figure 4, as well as a summary of different pre-processing steps used by prior studies given in Table 2, demonstrating large differences in the pre-processing implementation across studies.

## 7. UC Pre-Processing Techniques

Uterine contractions are commonly linked with FHR decelerations [94] and may provide vital information about the fetus during labour. This requires capturing the intensity, duration and shape of uterine contractions to make an assessment. However, the UC signals obtained from CTG often exhibit poor quality due to technical constraints due to the majority of signals being acquired using TOCO [87]. Clinical practice typically takes this into account by only utilising UC signals when they are reliable [37]. Therefore, for computerised CTG analysis methods to avoid utilising poor quality information, the majority of studies exclude UC signals from their approaches [95]. In limited studies such as [37,87], the raw UC signals are pre-processed by assessing the quality using an established autoregressive model [96]. Then, UC signals of poor quality are replaced by zeros before using them in the computerised CTG analysis. Therefore, pre-processing approaches for UC signals in the literature involve one of two methods: (1) the complete exclusion of UC signals, where they are not considered or analyzed in the study or (2) the replacement of poor-quality UC values with zeros, effectively removing or discounting unreliable or noisy data points from the analysis.

## 8. Fetal Compromise Classification Criteria

Before training computerised CTG analysis approaches, it is necessary to assign labels to the CTG data to represent the status of the fetus at birth. Different studies have used a variety of classification criteria. The predominant objective indicator of fetal compromise that has been used in the literature is the umbilical artery pH. The pH value indicates the degree of fetal acidemia resulting from the dispersion of accumulated lactic acid in the fetal circulation [26]. The fetal pH is typically measured from a sample, which may be acquired from fetal scalp, umbilical arterial or umbilical venous blood. As fetal carbon dioxide is removed through the umbilical arterial blood, it typically has a slightly higher pH reading than venous blood. When arterial pH is used to define the class labels, it is important that we only use the pH readings that are validated to prevent errors in the labels. An arterial pH value that is measured is considered valid if it is at least 0.02 more than the venous pH [25]. BDecf and Apgar scores are two other predictors of fetal compromise used in the existing literature. Typically, pH values below 7.05 and BDecf values above 10 mmol/L are strongly correlated with adverse neonatal outcomes [26,27], whereas a 5-min Apgar score of less than or equal to 6 indicates an increased risk of neonatal death [28]. Consequently, umbilical artery pH, BDecf and Apgar scores are widely employed as common clinical endpoints to define fetal compromise. In addition, Petroziello et al. [37] have used other fetal outcomes such as stillbirth, neonatal death, neonatal encephalopathy, neonatal intubation, and admission to neonatal intensive care for 48 h or more to define fetal compromise. Table 3 shows different classification criteria and thresholds used in the literature.

## 9. Automated Fetal Compromise Classification Methods

**Figure 5:**
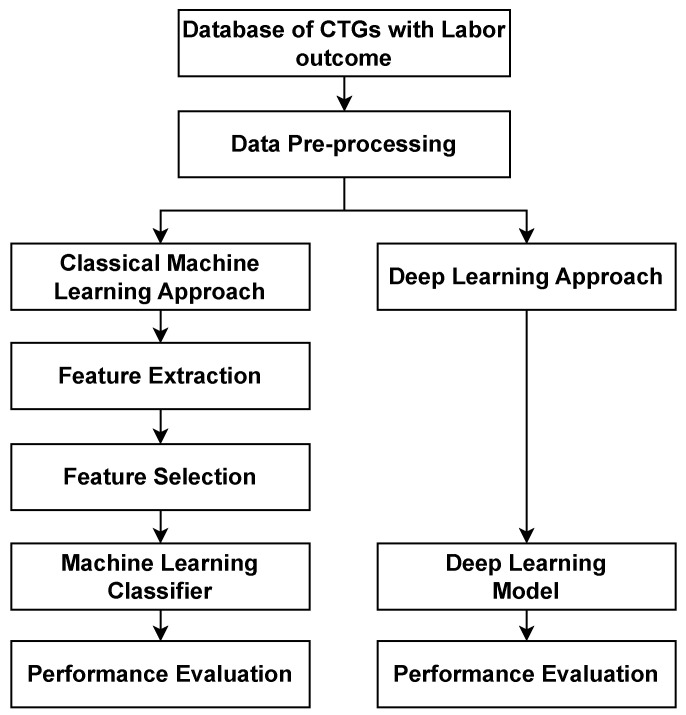
An overview of the steps for the computerised analysis of fetal compromise showing the classical machine learning approach and deep learning approach.

The development of automated fetal compromise classification approaches began with classical feature-based machine learning methods [35,36] and later evolved to modern deep learning methods [37,86]. Classical methods use additional steps like feature extraction and selection to identify the most relevant features from the raw CTG signals, while modern deep learning methods work directly on the raw CTG signals. In this section, we review the basis of each stream. Figure 5 provides an overview of how the pre-processing steps and classification methods work in sequence.

### 9.1. Feature Extraction

Feature extraction plays a crucial role in fetal compromise detection. This process is aimed at extracting relevant information or characteristics from the raw CTG signals. These extracted features serve as indicators of fetal health, allowing healthcare professionals or computerised methods to monitor and evaluate the condition of the fetus. The extracted features used in classification algorithms in existing studies can be broadly categorised into the following categories: (1) morphological and time domain, (2) frequency domain and (3) non-linear.

#### 9.1.1. Morphological and Time Domain

The basic morphological features defined by the FIGO guidelines for evaluating CTG signals are baseline, acceleration, deceleration and variability [11]. These are characterised by the structure of the FHR waveform and are easily visible to the naked eye. Therefore, these are routinely used by obstetricians and midwives in the visual assessment of CTGs in clinical practice [43]. Inspired by this, initial automatic classification systems used statistical techniques to compute new time domain features such as short-term variability (STV) and long-term irregularity (LTI), which quantify the variability in the FHR in the short term and long term, respectively, as well as the delta, total delta, mean and standard deviation of FHR [100].

#### 9.1.2. Frequency Domain

Frequency domain methods assess the spectral energy content in each frequency component of the FHR and use them as features. The range of the FHR signal in the frequency domain is often divided into four bands: very low frequency (VLF: 0–0.03 Hz, associated with a long time period and non-linear contributions), low frequency (LF: 0.03–0.15 Hz, related to fetal sympathetic nervous system activity), high frequency (HF: 0.5–1 Hz, reflecting fetal breathing) and movement frequency (MF: 0.15–0.5 Hz, correlated with fetal movements and maternal activity) [101]. The power spectral density is estimated in each band using Fourier transform, autoregressive or wavelet transform-based models [49]. Inspired by the usage of the ratio of LF/HF in the analysis of adult heart rate variability (HRV), a similar ratio computed as LF/(MF+HF) has been used as another fetal HRV feature as it quantifies the balance of parts of the fetal autonomic nervous system [102]. Although the frequency domain features can inspect periodic trends in heart rate variations, they are sensitive to artefacts and do not identify non-periodic trends in the FHR variations [49].

#### 9.1.3. Non-Linear Domain

Non-linear features were introduced to investigate and quantify non-periodic variations of the FHR signals. Almost all non-linear features used for FHR analysis are introduced from adult HRV research. Among them, the most frequently used are approximate entropy and sample entropy, which contain information on the fetal state [103], as well as Lempel Ziv Complexity, which is a non-linear feature used to examine the recurring patterns in a continuous signal [104]. Phase-rectified signal averaging is another non-linear method that is used to calculate the decelerative capacity of FHR. This feature quantifies the downward movement of FHR and has shown significantly better performance for acidosis prediction compared to STV [105]. Symbolic dynamics analysis, fractal analysis, detrended fluctuation analysis and Poincaré maps are other techniques used in the literature to compute other non-linear features for FHR analysis [49]. However, analysing FHR using these features has two major limitations: (1) the accuracy of these measures relies on the quality of the FHR signal and (2) a specific data length needs to be selected to obtain a reliable estimate of the values for non-linear features [106].

### 9.2. Feature Selection

All features calculated across these categories might not be as informative as expected and might contain overlapping information. Therefore, a feature selection or dimensionality reduction step is often used to determine the most influential features that contain useful information for classification. An optimal selection of features will in return improve the computational efficiency and potentially improve the discriminative capability of the classifier [35]. Principal component analysis (PCA) [107], information gain [95], and relevance in estimation features (RELIEF) [35] are feature selection methods often used in literature.

### 9.3. Classical Machine Learning Classifiers

Initially, research in automated fetal compromise detection was based on simple logic-based computer software algorithms that resembled the clinical decision-making process based on the FHR baseline, accelerations and deceleration [29]. Later, with the advancement of machine learning algorithms, these methods utilised feature extraction and feature selection steps prior to use in classical ML algorithms such as Bayesian models [108], support vector machines (SVM) [39,98,109], adaptive boosting (AdaBoost) [43], random forest (RF) [36,110], decision trees (DT) [43], deep Gaussian processes (DGP) [41], logistic regression (LR) [93] and artificial neural networks (ANN) [111,112] for detecting fetal compromise. The following sections summarise feature-based machine-learning methods used for fetal compromise detection.

#### 9.3.1. AdaBoost

Adaboost is an ensemble learning algorithm that combines multiple weak classifiers to create a strong classifier to reduce overfitting and improve performance. Spilka et al. [35] utilised the nearest mean classifier with AdaBoost to classify pathological cases of FHR using more than 50 features taken from morphological, frequency and non-linear domain features. This method utilised the RELIEF technique for feature selection and the synthetic minority oversampling technique (SMOTE) to tackle the class imbalance of the data before classification. Zhao et al. [43] proposed new software for computerised analysis of the FHR signal (CAS-FHR) that extracts 47 comprehensive features covering morphological, time, frequency and nonlinear domains. The same study compared decision trees, SVM and AdaBoost machine learning algorithms for assessing fetal state and found that AdaBoost had a stronger performance for classification.

#### 9.3.2. Artificial Neural Networks

Artificial neural networks are highly flexible and powerful models inspired by the human brain, capable of learning complex patterns and relationships in data through interconnected layers of neurons. Georgieva et al. [97] used six clinical features and six features from the FHR as input data to a feed-forward ANN. PCA was utilised for feature selection prior to using it as input to the ANN. Classifier performance was improved by averaging the output of 10 independently trained ANNs and testing on a larger dataset consisting of 7568 cases. Comert and Kocamaz [113] showed that ANNs gave better results in classifying FHR signals as hypoxic or normal compared to other ML algorithms like SVMs, extreme learning machines, radial basis function networks, and RFs. In another study, Comert and Kocamaz [111] analysed the effects of linear and non-linear features of FHR on the performance of detecting fetal compromise for three stages of labour. By using ANN as the classifier, they identified that using linear and non-linear features together gave the best performance and that the contribution of non-linear features was greatest for the second stage of labour.

#### 9.3.3. Bayesian Models

Bayesian models provide a probabilistic framework for modelling uncertainty and incorporating prior knowledge. Dash et al. [108] employed Bayesian models to classify fetal status based on selected FHR features and found that it demonstrated better performance compared to SVMs.

#### 9.3.4. Decision Trees

Decision trees are simple yet powerful algorithms that provide an interpretable and effective approach for fetal compromise detection. Zhao et al. [43] compared decision trees with other ML algorithms and demonstrated their efficacy in assessing fetal state. However, its performance was lower than the AdaBoost model compared in the same work.

#### 9.3.5. Deep Gaussian Processes

Deep Gaussian Processes are flexible models that can capture complex relationships while also providing uncertainty estimates to aid decision-making. Feng et al. [41] used a different approach by utilizing the UC signal with the corresponding FHR signal in DGPs to improve the performance of the classification of fetal well-being.

#### 9.3.6. Logistic Regression

Logistic regression is a linear classifier that can be used to model the relationship between input features and the probability of fetal compromise. O’Sullivan et al. [93] showed that including electronic health records and the duration of the last two stages of labour in addition to the CTG features improved the performance of the LR model in identifying fetal compromise.

#### 9.3.7. Random Forest

Random forest is an ensemble method that can combine multiple decision trees, providing robustness against overfitting and the ability to capture complex relationships. Spilka et al. [36] used latent class analysis to define classes and employed the RF algorithm for classification as it provides faster training than the boosting classifiers. Afridi et al. [110] also showed the effectiveness of using RF models for discriminating fetal status.

#### 9.3.8. SVM

Support vector machines are binary classifiers that find the best hyperplane to separate classes by maximizing the margin between them, allowing for effective handling of both linear and non-linear decision boundaries. Georgoulas et al. [114] proposed a novel method to extract scale dependant features of FHR signal using discrete wavelet transform (DWT) and used SVMs for classification. In [107], Georgoulas et al. used SVMs to predict the risk of metabolic acidosis using FHR features extracted from the time and frequency domain in addition to its morphological features. The experimental results of this work indicated that the SVM classifier with the radial basis function (RBF) kernel shows better classification performance than using SVMs with polynomial kernels. SVM is also used in [109] to detect normal or at-risk fetuses based on FHR features extracted through empirical mode decomposition. A different study by Stylios et al. [39] showed that the least square support vector machine (LS-SVM) can be used to discriminate compromised from healthy fetuses. In another study [44], LS-SVM showed increased classification accuracy of the fetal hypoxic assessment when features found using the novel approach called image-based time-frequency (ITBF) analysis were used. Similarly, sparse SVM classification managed to outperform clinical practice in fetal acidosis detection by selecting only a small number of features [98].

### 9.4. Deep Learning-Based Classifiers

Deep learning-based classifiers are powerful modern approaches that greatly benefit from the availability of well-defined, open-access databases as they allow for the learning of complex characteristics independently from the raw data without relying on the features derived from human knowledge. In prior works on automatic fetal compromise detection, the most typically used deep learning algorithms are convolutional neural networks (CNN) and long short-term memory networks (LSTM). CNNs can capture hidden characteristics of both spatial and temporal data, while LSTMs are capable of capturing both long- and short-term dependencies in time-series data [87].

These methods have high computational complexity during the training process, but once trained they can be configured to predict fetal compromise in real-time. However, to achieve this, deep learning models should be trained using diverse and large datasets for the model to generalise better. Otherwise, these methods can overfit the training data and perform poorly on unseen data. The following sections summarise the recent deep learning-based methods used for fetal compromise detection.

#### 9.4.1. Convolutional Neural Networks

CNNs are deep learning models specifically designed to automatically extract relevant features from raw data, enabling the detection of fetal compromise by capturing intricate patterns and spatial dependencies. One of the main benefits of CNNs is their ability to simplify the model through weight sharing and subsampling [115]. Nevertheless, CNNs primarily learn local features and lack knowledge about the global relationships within the data. Additionally, CNNs require a substantial number of training samples to enhance the model’s generalisation. In the domain of FHR classification, the effectiveness of 1D CNN has been demonstrated. This type of CNN can process a time series of FHR signal data, extracting features by applying various kernels to classify the signal as normal or compromised. Petrozziello et al. [87] compared both LSTM and CNN models using a dataset of over 35,000 labouring CTGs. The results indicated that both LSTM and CNN outperformed clinical practice in predicting fetal compromise and concluded that CNNs surpassed conventional classification methods based on feature extraction. Petrozziello et al. [37] further explored the use of a multimodal CNN (MCNN) for predicting fetal acidemia at birth. This approach employed a quality vector, UC, and FHR signals as inputs to the MCNN model. Their MCNN outperformed the current clinical practice and OxSys1.5 [34] prototype system in evaluating fetal compromise during labour. Additionally, a stacked MCNN was proposed, where the output from the first MCNN, computed on the last 60 min of the first stage of labour CTG signals, served as input to a second MCNN. The second MCNN also used the last 30 min of the second stage of labour CTG signals as input, ultimately providing the final classification outcome. However, the best performance was reported by the MCNN when analysed on the last 60 min of CTG signal regardless of labour. In a different study, Li et al. [116] divided a 20-min FHR segment into 10 window segments and used a CNN to process them in parallel. A majority voting technique was used to provide the final outcome. They showcased the superiority of the CNN approach by comparing it with a feature-based SVM and a multilayer perceptron classification. Zhao et al. [86] presented a different approach using a 2D CNN with 2D images obtained from continuous wavelet transform (CWT) to automatically predict fetal acidemia. Furthermore, the same authors utilised a 2D CNN with 2D images constructed using a recurrence plot (RP) to capture the non-linear characteristics of the FHR signals, aiming to predict fetal hypoxia.

#### 9.4.2. Long Short-Term Memory Networks

LSTMs are a type of recurrent neural network that can effectively model temporal dependencies in sequential data, making them suited for fetal compromise detection by capturing long-term dependencies and recognizing patterns over time. The study by Petrozziello et al. [87] examined CNN and LSTM as two standalone models and demonstrated that CNNs outperform LSTM in predicting fetal compromise. However, work by Liu et al. [46] shows that a novel attention-based method combining CNN and a bi-directional LSTM (CNN-BiLSTM) can achieve satisfactory performance in detecting fetal compromise. Its CNN-BiLSTM captures complex non-linear, spatial and temporal characteristics of the FHR, while the attention mechanism focuses on the important features of the input. However, this architecture showed overfitting, and DWT was used to obtain another feature to reduce the issue of overfitting.

A summary of computerised fetal compromise detection methods along with their strengths and limitations is presented in Table 4.

### 9.5. Performance Evaluation

Fetal compromise detection using CTG data suffers from a high imbalance in classes. For example, only about 7% of CTU-UHB traces fall under compromised cases when a pH threshold of 7.05 is used as the outcome measure. Several approaches like under-sampling the majority class [117], oversampling the minority class [35] and using class weights in the loss function [37] are used to tackle the disproportion in classes. Under-sampling is usually not desirable as it may result in the loss of important data [118].

When evaluating an ML or DL model, the whole dataset is separated into two sets: a training set and a test set. When only a single division of a training set and a test set is allocated for evaluation, this is called a holdout. Despite the faster evaluation of this method, the model performance has a significant bias towards the data points allocated to the test set and therefore will depend on how the division is made. Especially when smaller datasets are used, the generalizability of the trained model will be lower. Hence, to overcome this limitation k-fold cross-validation is typically used. Here, the dataset is divided into k subsets and the holdout method is repeated k times. At each time, one of the subsets is kept for testing while the remaining subsets are used for training. Finally, the average performance across all k trials is reported.

To measure the performance of computerised fetal compromise detection algorithms, various metrics are utilised in the literature. Traditional metrics like accuracy and error rate are not suitable for evaluating the performance of the classifiers on imbalanced data as they are sensitive to the distribution of the data. Alternative measures, including sensitivity, specificity, F-measure, Matthew’s correlation coefficient and geometric mean derived from the confusion matrix, are typically used to present their performance [118].

Sensitivity and specificity are preferred metrics as the objective of computerised fetal compromise detection is to maximise the number of the correctly predicted positive class while minimising the number of the incorrectly predicted negative class. However, sensitivity and specificity are often inversely correlated, so some trade-off in specificity has to be tolerated. Often, receiver operating characteristics (ROC) curves are formed by plotting true positive rates (equivalent to sensitivity) against false positive rates (equivalent to 100%—specificity) for different thresholds. These curves provide a graphical representation of the performance of the classifier across different sensitivity and specificity thresholds. A single metric called area under the curve (AUC) is computed after plotting the ROC to assess the performance of different classifiers. AUC values greater than 0.5 mean that the model is performing better than random chance, while an AUC value of 1.0 indicates perfect classification. A summary of the evaluation metrics used in the literature is given in Table 5. The performance of computerised CTG analysis methods according to these performance metrics is given in Table 6.

## 10. Discussion

This review highlights that a comprehensive understanding of the complex and heterogeneous nature of cardiotocography signals for detecting fetal compromise remains an open challenge. In addition to questions about the most relevant physiological features of CTG signals for interpreting fetal state, we draw attention to several additional technical challenges, as follows.

It can be seen that a common challenge faced by all computerised CTG analysis methods is the quality of the CTG data acquired. Petrozziello et al. [37] showed that quantification of signal quality is important and poor signal quality results in the adverse performance of the models. They achieved the current state-of-the-art AUC of 0.82 on the entire CTU-UHB database in fetal compromise prediction with pH threshold <7.05 by using a quality parameter as an input along with FHR and UC in their MCNN model. Although direct fECG provides superior signal quality, its invasiveness prevents its widespread use. The performance of the non-invasive Doppler ultrasound-based CTG and NI-fECG methods also differ significantly from the current gold standard of direct fECG measurement. According to prior studies, NI-fECG demonstrates a mean positive percent agreement with the fetal scalp electrode between 81 and 83%, compared to 62–73% for Doppler ultrasound CTG [61,62]. Despite the lower agreement, Doppler ultrasound-based CTG is still the most widely used monitoring technique. For instance, about 75% of the CTU-UHB dataset is acquired from Doppler ultrasound-based CTG. This raises questions of whether the performance of all computerised CTG analysis methods are impacted by the quality of Doppler ultrasound-based records. We suggest that future work should look at the viability of using only direct fetal ECG or NI-fECG records for training computerised CTG algorithms for fetal compromise prediction and compare these to ultrasound-based methods.

Further, it is important to note that the direct fetal ECG or NI-fECG terms in the context of these studies are more closely related to QRS detection and heart rate trends rather than true fECG, which entails more comprehensive cardiac waveform display and time intervals. Therefore, we suggest that future works may also evaluate the potential of the raw fECG signal and its features for detecting fetal compromise.

As highlighted by Table 3, different classification criteria like base deficit, Apgar scores, and umbilical cord pH are used to define the fetal compromise where the latter is the most widely used. According to [119], the umbilical artery base deficit is comparable or inferior to pH as a perinatal outcome measure. Further, the results of [119,120] show that low cord pH is strongly associated with poor perinatal outcomes and therefore is a strong candidate for verifying the performance of computerised CTG analyses. Different pH thresholds such as 7.05 [35,37,38,39,40], 7.10 [41,42], 7.15 [43,44,45,46] and 7.20 [47] have been used in existing studies to define the fetal compromised babies. This use of multiple pH thresholds hinders the comparison, improvement and criticism among different proposed methods. Therefore, agreeing on common criteria by the research community is essential to drive this field forward. As can be seen in Table 3, a pH threshold of 7.05 is the most widely used criterion to define fetal compromise. Therefore, we propose that future works should adopt this criterion as the standard to define fetal compromise and use it when reporting the performance of new computerised methods for fetal compromise detection.

As the performance of the algorithms depends on the data itself, pre-processing plays a large role in the comparison of different computerised analysis methods. Comparing different studies without a standard pre-processing procedure may lead to incorrect conclusions about whether a particular classifier or feature set is superior to a previously identified work. We have summarised the typical pre-processing steps in Figure 4, but there is yet no conclusive evidence of the optimum signal length, artefact removal, interpolation method, downsampling frequency or smoothing technique that should be used. Future work should try to address these questions to define a standard pre-processing methodology. Moreover, data manipulation, like data augmentation, should be carefully carried out such that no information is leaked into the test set. If this happens, the results will be subject to bias and the conclusions could be misleading [121]. For example, we believe that the performances achieved by the two studies [45,86] are likely not representative of real-world performance as the data is augmented using CWT and RP before the data is split into training and test sets. Therefore, care should be taken when pre-processing and splitting the data so that the independence of the test set is maintained.

Furthermore, existing analysis methods primarily use static and long segment lengths taken from the end of the CTG recording for detecting fetal compromise [37,89,98]. This results in predictions only made close to the time of delivery, leaving inadequate time for the clinicians to make clinical interventions. Therefore, studies such as [98] have suggested that future work should assess the evolution of FHR over time rather than evaluating a static segment. A method of this nature will be more clinically relevant as this could potentially detect fetal compromise events much earlier and enable clinical intervention.

In considering prediction at earlier time points, currently a single label is given to the overall CTG trace based on a clinical metric typically taken after birth (i.e., pH < 7.05), making it a ‘weak label’ where it is unclear whether a particular section of the CTG trace contains an abnormality or not. In particular, if it is assumed that the labels are correct, the information regarding the specific location of the abnormality on the CTG trace is not known. Therefore, there is a significant risk of introducing noisy labels unless the distress is chronic and prevails throughout the extent of the trace. However, different types of fetal hypoxia exist such as acute, subacute, evolving and chronic, and they generally occur in different forms [56]. Some cases show evidence of distress in the CTG but no acidemia in blood gas analysis. One reason could be the treatment paradox. A treatment paradox is when the strong predictor of a complication results in rapid intervention and improved outcomes as a result of effective treatment [122], thereby making the class labels unreliable. An approach to making the labels more reliable in theory would be to use clinical experts to annotate the CTGs. This consideration has led to the creation of two new sets of expert annotated labels for the CTU-UHB dataset, which are also available for public access [21,123]. Conversely, this would again lead to the primary issue of human bias in deciding the labels for each recording.

Another aspect studied by researchers is the usage of electronic health records (EHR) as input to the classifier. The study [93] used clinical variables, including gestation, parity, hypertension and the duration of stages of labour to show that the classifier performance can be enhanced by using EHR information. These records can provide valuable information on the baby and the mother, but it is essential that we only include health records that are known prior to or during labour because the end goal is the real-time detection of fetal compromise during labour.

In evaluating the performance of the computerised approaches for fetal compromise detection, various metrics have been used in the literature. However, the main objective of these methods should be to achieve a high sensitivity while maintaining high specificity. Since specificity decreases when sensitivity increases, a certain degree of specificity decrease has to be tolerated. The current clinical sensitivity or true positive rate (TPR) in detecting fetal compromise is about 31–48% at a 16–21% false positive rate (FPR) [34,37,124]. Therefore, the research community should agree to present their performance in terms of TPR at approximately 10–20% FPR such that it can be compared with current clinical performance. This then raises an important question of what would be a necessary level to use in clinical practice. According to Georgieva et al. [34], the performance should exceed 60% TPR at 15% FPR to gain tangible clinical benefits. Nevertheless, this is still debatable, and consensus must be reached to achieve clinical translation in the future.

As can be seen from Table 6, the current best performance achieved on the complete CTU-UHB dataset with pH threshold <7.05 is 33–65% TPR at 5–20% FPR by Petroziello et al. [37]. Although there are other studies that have reported higher performance, they have either used a different class definition criteria or have only used a subset of the dataset to report performance. A subset of the dataset is chosen by some studies [38,45] either to balance the distribution of classes or to remove records based on signal loss. We believe that the imbalanced nature of the compromised and normal classes should be accepted as it reflects the real world, and choosing a subset of data to balance the classes is not ideal. Rather, other means of tackling the class imbalance should be sought such as using class weights [37]. Furthermore, all studies should cross-validate their results on the complete CTU-UHB dataset such that the reported performances are generalised and unbiased. However, the validation on CTU-UHB is still small in terms of the number of recordings compared to large clinical studies as presented in Section 4, which range between 7730–46,042 participants [33,77].

Another possible reason for the low performance of computer-based approaches could be the limited number of public CTG datasets. Currently, accessible databases have a very low number of compromised cases and are typically taken from a single institution. The CTU-UHB with 552 raw FHR and UC records with pH values is available for public access as a benchmark dataset to evaluate and compare the performance of new algorithms. Even so, much larger, multi-centre datasets with well-documented outcomes, including obstetric clinical information, are needed for the automatic computer-based classification methods to be robust and reliable in clinical use [125]. This cannot be achieved by a single stakeholder, and the field should look to create collaborations between multinational and multidisciplinary research groups to solve this challenge collectively [126]. This will help further demonstrate the performance of these data-driven methods prior to prospective clinical validation. Furthermore, a consensus on the pre-processing steps, outcome measures and evaluation metrics must be reached to allow for transparency, reproducibility and comparison among different approaches.

## 11. Conclusions

To our knowledge, this is the first review to summarise the physiological basis for CTG, FHR and UC monitoring technologies; computerised CTG analysis systems in clinical practice; public and private CTG datasets; pre-processing steps; and classification methods used in automated data-driven approaches for fetal compromise detection. The quality of the CTG data is one of the main obstacles in achieving the performance required for widespread clinical translation. Therefore, further research on emerging monitoring technologies such as NI-fECG and EHG is warranted to improve the underlying data quality for building automated methods. We also believe that a standardised pre-processing workflow and criteria for the classification of fetal compromise would address criticisms that impact progress in this field while allowing for reproducibility and comparability. Furthermore, rather than working on private datasets, we recommend, where possible, making datasets public as advancing the performance of deep learning methods depends on large multi-center datasets to improve generalisability.

## Figures and Tables

**Table 1:** Overview of public and private CTG datasets.

Dataset	Signal Format	Outcome Data	Number of Recordings	Availability
UCI ML Repository [80]	FHR and UC features only	Three classes: Normal (N) Suspect (S) Pathological (P)	2126 N = 1655 S = 295 P = 176	Public
CTU-UHB Dataset [81]	Raw FHR and UC at 4 Hz	pH	552	Public
Lyon Dataset [38]	Raw FHR and UC at 10 Hz	pH	1288	Private
Oxford Dataset [37]	Raw FHR and UC at 4 Hz	pH	35,429	Private

**Table 2:** Overview of different FHR pre-processing techniques used in terms of segment selection, artefact removal, signal interpolation, downsampling and signal detrending.

Year	Authors	Segment Selection	Artefact Removal	Signal Interpolation	Downsampling Frequency	Smoothing and Detrending
2013, 2014	Spilka et al. [35,36]	Not specified	FHR < 50 bpm or FHR > 210 bpm corrected Long gaps > 15 s not included	Hermite spline	None	None
2016	Stylios et al. [39]	Last 30 min of 1st stage of labour	Not specified	Hermite spline	None	None
2017	Georgoulas et al. [40]	Not specified	FHR < 50 bpm or FHR > 200 bpm corrected Long gaps > 15 s not included	Hermite spline	None	None
2018	Cömert and Kocamaz [47]	Last 15 min of 2nd stage of labour	FHR < 50 bpm or FHR > 200 bpm corrected Long gaps > 15 s not included	Cubic Hermite spline	None	None
2018	Petrozziello et al. [87]	Last 60 min of labour	Not specified	Linear	None	None
2018	Zhao et al. [43]	Not specified	Adjacent 5 FHR values with variability < 10 bpm and signal with zeros for >10 s not included FHR ≤ 50 bpm or FHR ≥ 200 bpm corrected FHR values with differences in adjacent values exceeding 25 bpm corrected	Linear and spline	None	None
2018	Cömert et al. [44]	Last 30 min of 1st stage of labour	Outliers and artefacts corrected	Cubic spline	None	Detrending: 2nd order polynomial
2018	Cömert et al. [88]	Last part of the 1st stage of labour	Long gaps > 15 s not included Outliers corrected	Cubic Hermite spline	None	Smoothing: Median filter Detrending: Not specified
2019	Fuentealba et al. [92]	Last 25 min of labour	FHR < 50 bpm or FHR > 200 bpm corrected	Hermite spline	None	None
2019	Petrozziello et al. [37]	Last 60 min regardless of labour stage	Not specified	Linear	0.25 Hz	None
2019	Zhao et al. [45,86]	Not specified	FHR < 50 bpm or FHR > 200 bpm corrected Long gaps >15 s not included FHR values with differences in adjacent values exceeding 25 bpm are corrected	Linear and cubic spline	None	None
2021	Liang and Li [89]	Last 30 min of labour	FHR< 50 bpm or FHR > 200 bpm corrected Long gaps >15 s not included	Hermite spline	1 Hz	Smoothing: Median filter
2021	Liu et al. [46]	Last 20 min of FHR	FHR < 50 bpm or FHR > 200 bpm corrected Long gaps > 15 s not included FHR values with differences in adjacent values exceeding 25 bpm are corrected	Linear and Hermite spline	1 Hz	None
2021	O’Sullivan et al. [93]	Not specified	FHR < 50 bpm or FHR > 210 bpm are corrected Patients with more than 30% traces missing are removed from the study FHR values with a change greater than 30% from the moving average are corrected	Not specified	None	None

**Table 3:** Overview of different classification criteria used to define classes and their corresponding class distribution.

Year	Authors	Datasets	Classification Criteria (Denoting All Unhealthy Classes as Compromised and Healthy Classes as Normal)	Number of Recordings
2013	Spilka et al. [35]	CTU-UHB	pH ≤ 7.05 as Compromised	Total = 552 Normal = 508 Compromised = 44
2013	Georgieva et al. [97]	Subset of Oxford Dataset	Training: 7.27 < pH < 7.33 as Normal pH < 7.1 as Compromised Testing: 7.22 < pH < 7.27 as Normal pH < 7.1 as Compromised	Training set: Total = 124 Normal = 62 Compromised = 62 Testing set: Total = 252 Normal = 126 Compromised = 126
2016	Spilka et al. [38]	Lyon Dataset CTU-UHB	pH ≤ 7.05 as Compromised	Training: Lyon DB Total = 1288 Normal = 1251 Compromised = 37 Testing: CTU-UHB Total = 420 Normal = 400 Compromised = 20
2016	Stylios et al. [39]	CTU-UHB	pH ≤ 7.05 as Compromised	Total = 552 Normal = 508 Compromised = 44
2017	Georgoulas et al. [40]	CTU-UHB	pH ≤ 7.05 as Compromised	Total = 552 Normal = 508 Compromised = 44
2017	Spilka et al. [98]	Lyon Dataset	pH ≤ 7.05 as Compromised	Total = 1288 Normal = 1251 Compromised = 37
2018	Cömert and Kocamaz [47]	CTU-UHB	pH < 7.2 as Compromised	Total = 552 Normal = 375 Compromised = 177
2018	Feng et al. [41]	CTU-UHB	pH > 7.2 as Normal pH < 7.1 as Compromised	Total = 447 Normal = 358 Compromised = 62
2018	Petrozziello et al. [87]	Oxford Dataset	pH < 7.05 as Compromised	Total = 35,429 Normal = 33,959 Compromised = 1470
2018	Zhao et al. [43]	CTU-UHB	pH < 7.15 as Compromised	Total = 552 Normal = 447 Compromised = 105
2018	Cömert et al. [44]	CTU-UHB	pH ≤ 7.15 as Compromised	Total = 552 Normal = 439 Compromised = 113
2018	Cömert et al. [88]	CTU-UHB	pH ≤ 7.15 as Compromised	Total = 552 Normal = 439 Compromised = 113
2019	Fuentealba et al. [92]	CTU-UHB	pH > 7.2 and BDecf < 12 as Normal pH < 7.05 and BDecf ≥ 12 as Compromised	Total = 372 Normal = 354 Compromised = 18
2019	Petrozziello et al. [37]	Oxford Dataset CTU-UHB Lyon Dataset	Normal: pH ≥ 7.15 Severe Compromise: pH < 7.05 and a composite outcome of stillbirth; neonatal death; neonatal encephalopathy; intubation or cardiac massage followed by admission to neonatal intensive care for ≥ 48 h Moderate Compromise: pH < 7.05 Intermediate: 7.05 ≤ pH < 7.15	Oxford Dataset Training: 30,115 Testing: 4429 Normal = 4249 Moderate/Severe compromise with pH < 7.05 = 180 Testing: CTU-UHB Total = 552 Normal = 512 Compromised = 40
2019	Zhao et al. [86]	CTU-UHB	pH ≥ 7.15 as Normal pH < 7.15 as Compromised	Normal = 447, Compromised = 105 After CWT: Normal = 2682 Compromised = 630
2019	Zhao et al. [45]	CTU-UHB	pH < 7.15 as Compromised	Normal = 105, Compromised = 105 After RP 2D: Normal = 21,000 Compromised = 21,000
2020	Furuya et al. [99]	Private	5-min Apgar score < 8 or pH < 7.1 as Compromised	Total = 1301 Normal = 1184 Compromised = 117
2021	Liang and Li [89]	CTU-UHB	pH ≤ 7.05 as Compromised	Total = 552 Normal = 508 Compromised = 44
2021	Liu et al. [46]	CTU-UHB	pH ≤ 7.15 as Compromised	Total = 552 Normal = 439 Compromised = 113
2021	O’Sullivan et al. [93]	CTU-UHB	pH ≥ 7.15 and Apgar 5 ≥ 9 as Normal pH ≤ 7.0 or Apgar 5 ≤ 6 as Compromised	Total = 333 Normal = 310 Compromised = 23

**Table 4:** Summary of computer-based fetal compromise detection methods and their strengths and limitations.

Computerised Method		Strengths	Limitations	Computational Cost
Features	Morphological and Time Domain	Macroscopic features suitable for visual inspection Recognised clinical value for several features	Some features based on statistical computation with no direct link to fetal physiology	NA ${}^{*}$
	Frequency Domain	Capture periodic trends in FHR variations	Difficult to observe via visual inspective Sensitive to artefacts Does not identify non-periodic trends in FHR variations	
	Non-Linear	Quantify complex non-periodic variations of FHR	Sensitive to artefacts Values depend on the choice of parameters Some highly depend on the FHR signal length	
Classical Machine Learning		Internal operation is more easily understandable	Human involvement needed for feature extraction and selection	Low–High
ML Classifiers	Adaboost	Reduces the risk of overfitting by combining multiple weak classifiers	Computationally expensive due to the iterative nature of the algorithm	Medium
	ANN	Learns complex relationships among features	Prone to overfitting when a higher number of layers used	Medium
	Bayesian Models	Can incorporate prior knowledge and domain expertise through prior distributions	Computationally expensive for large datasets	High
	Decision Trees	Easy to interpret and visualise	Prone to overfitting if not properly regularised	Low
	Deep Gaussian Processes	Can learn complex, non-linear relationships	Can be challenging to interpret and visualise Computationally expensive for large datasets	High
	Logistic Regression	Simple and efficient Provides probabilistic outputs useful for interpretation	May not capture complex interactions between features	Low
	Random Forest	Robust against overfitting due to ensemble of decision trees	Computationally expensive for large datasets	Medium
	SVM	Effective in high-dimensional spaces Works well on small datasets Can handle linear and non-linear decision boundaries	Can be sensitive to the choice of kernel function and hyperparameters Computationally expensive for large datasets	Medium
Deep Learning		Feature extraction and selection are not needed Learns complex features from raw data	Lack of interpretability and transparency of operation High computational complexity	High
DL Classifiers	CNN	Reduces the complexity of the model by weight sharing and subsampling	Does not learn global relationships Requires more training samples for generalisation	High
	LSTM	Widely used for time series forecasting Learns temporal features	Takes longer time to train Requires more training samples for generalisation	Higher

* NA = Not applicable.

**Table 5:** Summary of evaluation metrics used in the literature.

Metric Name	Equation	Description
Accuracy (Acc)	$Acc=\frac{TP+TN}{TP+TN+FP+FN}$	The simple ratio between the number of correctly predicted points to the total number of points (probability of correct predictions) Not suitable for imbalanced datasets
Sensitivity (Se)	$Se=\frac{TP}{TP+FN}$	The proportion of the correctly predicted positive instances from the total positive instances
Specificity (Sp)	$Sp=\frac{TN}{TN+FP}$	The proportion of the correctly predicted negative instances from the total negative instances
Precision	$precision=\frac{TP}{TP+FP}$	The proportion of the correctly predicted positive instances from the total classified positive instances
Geometric mean (g − mean)	$g-mean=\sqrt{Se\times Sp}$	Measure of the balance between classification performances in both the majority and minority classes
Harmonic mean (F − measure)	$F-measure=\frac{2\times Se\times precision}{Se+precision}$	A measure of the effectiveness of classification
Matthew’s correlation coefficient (MCC)	$MCC=\frac{TP\times TN-FP\times FN}{\sqrt{\left(TP+FP)\times (TP+FN)\times (TN+FP)\times (TN+FN\right)}}$	Minimally influenced by imbalanced data, the correlation coefficient between the observed and predicted classifications (range from −1 to +1), +1: perfect prediction 0: no better than random prediction −1: worst prediction
Area under the receiver operating characteristic curve (AUC)	Plot of the true positive rate vs. the false positive rate at all possible thresholds	Higher the AUC, the better the performance of the model at distinguishing between the classes Used to compare and evaluate different classification algorithms

**Table 6:** Performance of computer-based approaches for fetal compromise detection on the open-source CTU-UHB dataset. Performance is reported per classifier type when multiple approaches are used in a single work.

Year	Authors	Training Method ${}^{†}$	Sensitivity (%)	Specificity (%)	AUC	Independent Test/Train Data	Complete CTU-UHB at Threshold pH < 7.05 or pH ≤ 7.05
2013	Spilka et al. [35]	ID: FHR features augmented by SMOTE CT: Nearest mean classifier with AdaBoost CV: 44-fold	64.00	65.00	Not specified	Yes	Yes
2014	Spilka et al. [36]	ID: FHR features CT: RF CV: 2-fold repeated 5 times	72.00	78.00	Not specified	Yes	No, subset of dataset used and labelled using clinical annotations.
2016	Cömert and Kocamaz [112]	ID: FHR features CT: ANN CV: 5-fold	88.70	85.10	Not specified	Yes	No, subset of 100 records randomly chosen.
2016	Cömert and Kocamaz [111]	ID: FHR features CT: ANN, for 3 stages of labour CV: 10-fold	I = 95.89 II = 87.06 III = 85.87	I = 74.75 II = 75.90 III = 72.73	Not specified	Yes	No, complete dataset used but labelled using clinical annotations.
2016	Spilka et al. [38]	ID: FHR features CT: Sparse SVM CV: Train—Lyon Dataset, Test—CTU-UHB	40.00	86.00	0.79	Yes	No, subset of dataset which has less than 50% signal loss.
2016	Stylios et al. [39]	ID: FHR features CT: LS SVM with RBF kernel CV: 44-fold repeated 15 times	68.50	77.70	Not specified	Yes	Yes
2017	Georgoulas et al. [40]	ID: FHR features CT: LS SVM CV: 44-fold	72.12	65.30	Not specified	Yes	Yes
2018	Cömert and Kocamaz [47]	ID: FHR features CT: ANN, SVM, k-NN CV: 10-fold repeated 30 times	ANN = 68.52 SVM = 76.83 k-NN = 53.28	ANN = 70.29 SVM = 78.27 k-NN = 66.80	ANN = 0.76 SVM = 0.84 k-NN = 0.64	Yes	No, complete dataset used but with compromise defined by pH < 7.2.
2018	Feng et al. [41]	ID: FHR and UC features CT: Supervised two-layer DGP network CV: No CV but repeated 5 times	FHR = 73.00 FHR+UC = 91.00	FHR = 91.00 FHR+UC = 82.00	Not specified	Yes	No, subset of dataset used with compromise defined by pH < 7.1.
2018	Petrozziello et al. [87]	ID: Raw FHR and UC CT: CNN, LSTM CV: Train—Oxford dataset, Test—CTU-UHB	Not specified	Not specified	CNN = 0.82 LSTM = 0.81	Yes	Yes
2018	Zhao et al. [43]	ID: FHR features CT: AdaBoost CV: 10-fold CV	92.00	90.00	0.91	No, unclear whether data samples have been used multiple times.	No, complete dataset used with compromise defined by pH < 7.15.
2018	Cömert et al. [44]	ID: FHR features CT: LS SVM with RBF kernel CV: 10-fol repeated 100 times	63.45	65.88	0.65	Yes	No, complete dataset used with compromise defined by pH < 7.15.
2018	Cömert et al. [88]	ID: FHR features CT: SVM with RBF kernel CV: 10-fold repeated 30 times	57.42	70.11	Not specified	Yes	No, complete dataset used with compromise defined by pH < 7.15.
2019	Petrozziello et al. [37]	ID: Raw FHR and UC, FHR quality score CT: MCNN, stacked MCNN CV: Train—Oxford dataset, Test—CTU-UHB	MCNN * 33.00 48.00 58.00 65.00 Stacked MCNN * 33.00 45.00 58.00 65.00	MCNN * 95.00 90.00 85.00 80.00 Stacked MCNN * 95.00 90.00 85.00 80.00	MCNN = 0.81 Stacked MCNN = 0.82	Yes	Yes
2019	Zhao et al. [86]	ID: Raw FHR transformed to 2D using CWT CT: 8-layer CNN CV: 10-fold	98.22	94.84	0.97	No, data augmented before data split.	No, complete dataset used with compromise defined by pH < 7.15.
2019	Zhao et al. [45]	ID: Raw FHR transformed to 2D image using RP CT: 8-layer CNN CV: 10-fold	99.29	98.1	0.98	No, data augmented before data split.	No, subset of dataset used with compromise defined by pH < 7.15.
2021	Liang and Li [89]	ID: Raw FHR CT: CNN based on a weighted voting mechanism CV: No CV but holdout test set	80.93	79.85	0.90	Yes	No, performance is reported on a small holdout set of the dataset.
2021	Liu et al. [46]	ID: Raw FHR CT: CNN + BiLSTM + Attention + DWT CV: 10-fold repeated 10 times	75.23	70.82	Not specified	Yes	No, complete dataset used with compromise defined by pH < 7.15.
2021	O’Sullivan et al. [93]	ID: FHR and UC features, EHR, duration of stage I and II labour CT: Logistic regression CV: 5-fold	82.60	77.70	0.81	Yes	No, subset of dataset used with compromise defined by pH < 7.0 or Apgar ≤ 6.

${}^{†}$ ID = input data, CT = classifier type and CV = cross validation. * Multiple sensitivity/specificity pairs given.

## Data Availability

Not applicable.
